# Are the Poor Dying Younger in Malaysia? An Examination of the Socioeconomic Gradient in Mortality

**DOI:** 10.1371/journal.pone.0158685

**Published:** 2016-06-30

**Authors:** Jeevitha Mariapun, Noran N. Hairi, Chiu-Wan Ng

**Affiliations:** Julius Centre University of Malaya, Department of Social and Preventive Medicine, Faculty of Medicine, University of Malaya, 50603, Kuala Lumpur, Malaysia; University of Westminster, UNITED KINGDOM

## Abstract

**Introduction:**

Socioeconomic inequalities in health represent unfairness in the health distribution of a population. Efforts to produce information on mortality distributions in many low and middle income countries (LMICs) are mostly hampered by lack of data disaggregated by socioeconomic groups. In this paper we describe how mortality statistics obtained from multiple data sources were combined to provide an evaluation of the socioeconomic distribution of mortality in Malaysia, a LMIC located in the Asia Pacific region.

**Methods:**

This study has an ecological design. As a measure of socioeconomic status, we used principal component analysis to construct a socioeconomic index using census data. Districts were ranked according to the standardised median index of households and assigned to each individual in the 5-year mortality data. The mortality indicators of interest were potential years of life lost (PYLL), standardised mortality ratio (SMR), infant mortality rate (IMR) and under-5 mortality rate (U5MR). Both socioeconomic status and mortality outcomes were used compute the concentration index which provided the summary measure of the magnitude of inequality.

**Results:**

Socially disadvantaged districts were found to have worse mortality outcomes compared to more advantaged districts. The values of the concentration index for the overall population of the Peninsula are *C* = -0.1334 (95% CI: -0.1605 to -0.1063) for the PYLL, *C* = -0.0685 (95% CI: -0.0928 to -0.0441) for the SMR, *C* = -0.0997 (95% CI: -0.1343 to -0.0652) for the IMR and *C* = -0.1207 (95% CI: -0.1523 to -0.0891) for the U5MR. Mortality outcomes within ethnic groups were also found to be less favourable among the poor.

**Conclusion:**

The findings of this study suggest that socioeconomic inequalities disfavouring the poor exist in Malaysia.

## Introduction

Mortality statistics, especially mortality of infants and children under five years, have been used universally as important indicators of population health [1]. However, official mortality statistics generally report country-level rates and few countries are able to report mortality distributions across socioeconomic groupings within their populations [2].Information on such distributions are useful to inform country progress towards reduction of health disparities since it is known that the socioeconomically less advantaged have lower possibilities of attaining their full health potential and as a consequent, are not only at higher risk of dying but also of dying younger than those who are better-off [3,4]. Reducing such inequities in health is one of the aims of the recently launched Sustainable Development Goals (SDGs) [5]. In particular, the SDGs urge countries to work towards universal health coverage (UHC) as the main health sector vehicle to promote more equitable distributions of health.

Efforts to produce information on mortality distributions are mostly hampered by lack of data disaggregated by socioeconomic groups. Such data, especially those related to maternal and child deaths, have been collected for low and middle income countries (LMICs) funded to participate in the Demographic Health Surveys and the Multiple Indicator Cluster Surveys and as a result these countries have been able to produce evidence of socioeconomic gradient in mortality for their countries [6,7]. However, not all LMICs participate in these surveys and would thus need to look for other data sources to provide similar mortality evaluations. Malaysia, an upper middle income country in the Asia Pacific region is one such country and in this paper we describe how mortality statistics obtained from the country’s mandatory death registration system were combined with information obtained from population census and other routinely collected administrative data sources to provide an evaluation of the socioeconomic distribution of mortality in the country.

Malaysia provides a unique setting for a study on mortality distributions. A former British colony, the country achieved independence in 1957 and since then has made great strides to improve the social and economic well-being of its population. Notable achievements include a reduction in poverty rates from 49.3% of households in 1970 to 0.6% in 2014 and an increase in literacy rates among adults aged 15 to 24 years from 95.5% in 1982 to 98.2% in 2014 [8]. The country has also expanded the taxation-based public health care delivery system to provide access to comprehensive affordable health care services [9]. As a result, many of the country’s health indicators in 2013, including the life expectancies at birth for males of 72 years and for females of 76 years as well as infant mortality rates of 7.2 per 1000 livebirths, are comparable to countries at higher levels of economic development [10].

However, the fruits of economic development in Malaysia may not have been equally distributed to the entire population. Geographically, the country is made up of two equal land masses, divided by the South China Sea. The economically more developed and more populous half is located on the Malay Peninsula whilst the remaining half, consisting of the states of Sabah and Sarawak, is located on the northern part of the island of Borneo. The country’s population is made up of several ethnic groups with the Malays being the largest group (50.1% of the total population in 2010) followed by the Chinese (22.6%) and the Indians (6.7%) [11]. The rest of the population is made up of indigenous natives and non-citizens. In terms of wealth, the Chinese is deemed to be the most advantaged, ahead even of the politically powerful and more numerous Malays. Since the early 1970’s, the country has put in place several policies to assist the economically disadvantage Malays and indigenous natives. These include preferential opportunities for employment, land and house ownership [12]. Despite these measures, income disparities still exist between and within ethnic groups in the country [13,14]. Thus, this paper also has a secondary objective to examine if unequal wealth distribution between ethnic groups in Malaysia contributed to socioeconomic disparities in mortality in the country.

## Methods

### Source of mortality data

It is a legal requirement for all deaths occurring in the country to be registered with Malaysian authorities [15]. The national statistical agency, the Department of Statistics Malaysia (DOSM) is then responsible to collate and maintain the national mortality dataset. This dataset contains among others, information on age of the deceased as well as gender, ethnicity, nationality, date of death, residential address and cause of death. The cause of death may or may not be certified by a medical professional. In the latter situation, deaths are usually certified by the police or village chiefs and thus, are unlikely to record the actual biological cause of death. Approximately half of all deaths annually have not been certified by medical professionals. Due to the time required for compiling and cleaning, there is a lag-period of several years before the DOSM allows researchers access to the dataset.

For this study, we obtained complete anonymised mortality data for the years 1998 to 2006, which were the latest publicly available digitalised datasets. Table 1 shows the distribution of deaths by regions, namely Peninsular Malaysia, Sabah and Sarawak, for the years 1998 to 2006. In general, mortality rates show a decreasing trend from 1998 to 2006. It has been previously noted that death registration, though mandatory, was not complete for Sabah and Sarawak since the interiors of these two states are difficult to access due to mountainous forested terrains and lack of roads [16]. This is reflected in the mortality rates for these two states which are generally lower than that for the Peninsular. These same geographical challenges also act as barriers for the communities living in the interior of these two states to access health care, other public amenities and economic opportunities. Against this backdrop, the lower mortality rates recorded for Sabah and Sarawak compared to the Peninsular Malaysia do not appear to reflect the true mortality situation in the two states. In view of this, subsequent analysis has been restricted to examination of the mortality distributions in the Peninsular Malaysia.

**Table 1:** Number of Deaths and Mortality Rates by Region, Malaysia 1998 to 2006.

Region						Year				
		1998	1999	2000	2001	2002	2003	2004	2005	2006
	Number of Deaths									
Malaysia	Overall	106166	112,452	105370	104609	105982	111,644	112,700	113,714	115,084
	< age of 1	5572	4867	3586	2953	3126	3148	3105	3112	2877
	< age of 5	7343	6500	4872	4424	4252	4158	4044	4010	3652
Peninsular	Overall	91226	96,336	89180	90243	91523	96,378	96,901	98,125	99,286
	< age of 1	4618	3882	2943	2642	2762	2788	2757	2723	2485
	< age of 5	6150	5215	4038	3928	3764	3665	3583	3488	3141
Sabah	Overall	6578	6894	7031	5512	5833	6092	6444	6406	6392
	< age of 1	619	653	368	95	127	117	117	138	129
	< age of 5	742	793	452	153	172	161	161	187	181
Sarawak	Overall	8362	9222	9159	8854	8626	9,174	9,355	9,183	9,406
	< age of 1	335	332	275	216	237	243	231	251	263
	< age of 5	451	492	382	343	316	332	300	335	330
	Mortality Rate									
Malaysia	CDR	475.37	490.85	448.48	435.64	432.11	445.71	440.56	435.22	431.99
	IMR	10.61	9.32	6.67	5.84	6.32	6.54	6.44	6.56	6.09
	U5MR	13.99	12.45	9.06	8.75	8.60	8.64	8.39	8.45	7.73
Peninsular	CDR	510.86	526.48	475.78	471.28	468.14	482.90	475.61	471.76	468.31
	IMR	10.89	9.22	6.70	6.46	6.87	7.12	7.06	7.12	6.50
	U5MR	14.50	12.39	9.20	9.61	9.36	9.36	9.17	9.12	8.22
Sabah	CDR	263.75	266.51	262.39	200.81	207.64	211.84	218.86	212.46	207.40
	IMR	11.31	12.00	7.24	1.88	2.67	2.55	2.45	2.84	2.70
	U5MR	13.56	14.57	8.90	3.03	3.61	3.50	3.38	3.84	3.79
Sarawak	CDR	421.88	455.52	442.14	417.72	398.10	414.31	413.44	397.09	398.98
	IMR	7.27	7.13	5.74	4.69	5.27	5.51	5.30	5.80	6.13
	U5MR	9.79	10.57	7.97	7.44	7.03	7.53	6.89	7.75	7.69

CDR, Crude Death Rate; IMR, Infant Mortality Rate; U5MR, Under-5 Mortality Rate; CDRs are per 100,000 population; IMRs and U5MRs are per 1000 live births.

### Indicator of Socioeconomic Status of the Deceased

Commonly used indicators of a person’s socioeconomic status such as education level, occupation or income were not collected at time of death and thus were not available in the national mortality datasets. The Peninsular Malaysia is made up of nine states and two federal territories which are further broken down to 82 administrative districts. We constructed a district level index of socioeconomic status using household information collected during the 2000 Population and Housing Census of Malaysia. National population censuses are conducted by the DOSM once every 10 years. The 2000 census was chosen as this was carried out closest in time to the available mortality datasets.

The index was constructed using principal component analysis using 30 variables from the 2000 census which included quality of dwelling, access to utilities and infrastructure, ownership of durable consumer goods, house ownership as well as the education level and employment status of the household head. The median of the standardised socioeconomic index for each district was then matched with individual deaths in the mortality data based on the districts of residence of the deceased. Therefore, the socioeconomic status linked to the deceased is the average wealth of households in his/her district. Districts were then ranked and classified into socioeconomic quintiles according to the standardised median index of all households in Peninsular Malaysia. These socioeconomic quintiles were population weighted. The supporting information provides more details of the census data, the distribution of the socioeconomic index by states and the validity of the index (S1 Appendix).

### Mortality Indicators

Since the district socioeconomic index was developed using data from the census carried out in 2000 and to account for fluctuations in deaths across the years, the mortality data were averaged across five years, 1998 to 2002, for each district. Thereafter, four mortality indicators were used as outcome measures in this study; namely potential years of life lost (PYLL) per 1000 person years, standardised mortality ratio (SMR), infant mortality rate per 1000 live births (IMR) and under-five mortality rate per 1000 live births (U5MR).

We used the PYLL to identify districts in Peninsular Malaysia with higher burden of mortality at younger ages. For the computation of PYLL, contemporaneous life expectancies for males and females from age 0 to 80 years were obtained from annual life tables from 1998 to 2002. The PYLL for a deceased was calculated as contemporaneous life expectancy minus the age at death minus 0.5 years, following the assumption that death occurred mid-way between the last two birthdays [17]. The annual PYLL for all deaths in each district from 1998 to 2002 were averaged to account for fluctuations in deaths across the years. This average district PYLL was then divided by respective district population for the year 2000 to obtain the district PYLL rate. Most of our analyses focus on PYLL for the reason that it incorporates the aspects covered by standardised mortality and child mortality; given that it adjusts for gender and the age distribution and provides higher significance to younger deaths respectively.

We used the SMR to adjust for the effect of differences in age and gender between the populations of the districts in Peninsular Malaysia. The district SMR is the ratio of the total number of deaths in a given district over the deaths that would have been expected in the district if the age and gender-specific rates of the population of Peninsular Malaysia in the census year 2000 had applied. Age was controlled for using five-year age-bands from 0 to 80 years of age (0–4, …, 75–79, 80+) and the rates were also stratified by gender. The SMR is a form of indirect standardisation. Direct standardisation was not performed because of instability of district mortality rates due to low death counts within many of the younger age groups. Following the ethnic differences in the socioeconomic distribution, besides being adjusted for age and gender, the SMR was adjusted for ethnicity as well.

The IMR and U5MR were assessed because child mortality is an important indicator of development in health of a country. The IMR measures deaths of infants below the age of one per 1,000 live births and U5MR measures deaths of children below the age of five per 1,000 live births. All the district population estimates required for the estimations of the mortality indicators were obtained from the DOSM.

### Measurement of Inequality

As a measure of inequality, concentration indices were generated using STATA version 12.1 (StataCorp, Texas, USA) The concentration index (*C*) provides a summary measure of inequality in the socioeconomic distribution of mortality in the population [18]. This index ranges between the values of -1 and 1 [19], where an index of zero indicates no socioeconomic inequality in mortality between the districts, a negative index indicates that mortality is concentrated among the poorer districts and a positive index indicates that mortality is concentrated among the richer districts. The concentration indices were calculated using a convenient regression method, where the mortality indicators, were transformed on the fractional rank in the distribution of the districts which in turn were ranked by the district socioeconomic index. Standard errors were corrected for autocorrelation and heteroscedasticity using the Newey-West variance-covariance matrix. The 95% confidence intervals were calculated by first, multiplying standard errors by 1.96 and then adding or subtracting them by the concentration index.

In order to provide clearer visual distinction of the mortality distributions, the mortality indicators were mapped onto the map of districts in Peninsular Malaysia using ArcMap, a geospatial processing program under ArcGIS.

### Ethical Approval

All individual level data acquired for this study were anonymised data obtained fromDOSM. This study has been approved by the Medical Ethics Committee, University of Malaya Medical Centre, Malaysia.

## Results

Table 2 shows the distribution of the population in Peninsular Malaysia by socioeconomic quintiles. Malays made up most of the population in each quintile but the Malay population shares decrease from the 87.3% in the poorest quintile to 43.1% in the richest quintile. The opposite pattern was observed for the Chinese and Indians where their population shares increased from the poorest to richest quintile. Even so, the Malays still constituted the largest ethnic group even in the richest quintile.

**Table 2:** District, Population Totals and Ethnic Groups by Socioeconomic Quintiles.

		Socioeconomic Quintiles^1^				
	Peninsular	Poorest	Q2	Q3	Q4	Richest
Districts (n)	82	34	22	18	3	5
Population (in thousands)	18744.1	3996.2	3563.8	4268.5	3334.6	3581.0
Ethnicity (%)	Malay	3488.7 (87.3)	2255.9 (63.3)	2206.8 (51.7)	1297.2 (38.9)	1543.4 (43.1)
	Indigenous Natives	52.0 (1.3)	39.2 (1.1)	42.7 (1.0)	33.3 (1.0)	25.1 (0.7)
	Chinese	267.7 (6.7)	880.3 (24.7)	1293.4 (30.3)	1430.5 (42.9)	1375.1 (38.4)
	Indian	63.9 (1.6)	245.9 (6.9)	533.6 (12.5)	346.8 (10.4)	447.6 (12.5)
	Others	40.0 (1.0)	7.1 (0.2)	17.1 (0.4)	20.0 (0.6)	21.5 (0.6)
	Non Citizens	83.9 (2.1)	135.4 (3.8)	175 (4.1)	206.7 (6.2)	168.3 (4.7)

^1^Population weighted

Table 3 shows that mortality is concentrated among the poor in Peninsular Malaysia. The risk of dying is comparative higher among those in the poorest quintile compared to those in the richest quintile where mortality risks are 12% higher and 16% lower than the overall population in Peninsular Malaysia in the poorest and richest quintile respectively. The values of PYLL, IMR and U5MR for the poorest quintile are nearly double that for the richest quintile. The pro-poor mortality distribution is confirmed by the negative values of the concentration indices indicating that deaths are disproportionately concentrated in socioeconomically deprived areas. The highest extent of inequality was observed for PYLL and this was mainly due to the high concentration of child deaths among the poor.

**Table 3:** Distribution of mortality indicators by socioeconomic quintiles.

	Mortality Indicators (95% Confidence Intervals)			
	PYLL	SMR	IMR	U5MR
**Socioeconomic Quintiles**				
Poorest	122.15 (117.43 to 126.87)	1.12 (1.08 to 1.16)	10.10 (9.22 to 10.98)	14.93 (13.01 to 16.85)
2	116.45 (109.73 to 123.17)	1.12 (1.09 to 1.15)	8.92 (8.30 to 9.54)	12.33 (11.55 to 13.12)
3	97.16 (88.47 to 105.84)	1.00 (0.94 to 1.05)	8.06 (7.34 to 8.77)	11.22 (9.52 to 12.92)
4	84.61 (74.20 to 95.03)	0.97 (0.85 to 1.08)	7.40 (5.98 to 8.82)	9.40 (7.98 to 10.82)
Richest	69.55 (45.45 to 93.65)	0.84 (0.68 to 1.00)	6.00 (4.76 to 7.24)	8.20 (6.58 to 9.82)
**Concentration index**	-0.1334 (-0.1605 to -0.1063)	-0.0685 (-0.0928 to -0.0441)	-0.0997 (-0.1343 to -0.0652)	-0.1207 (-0.1523 to -0.0891)

**Fig 1:**
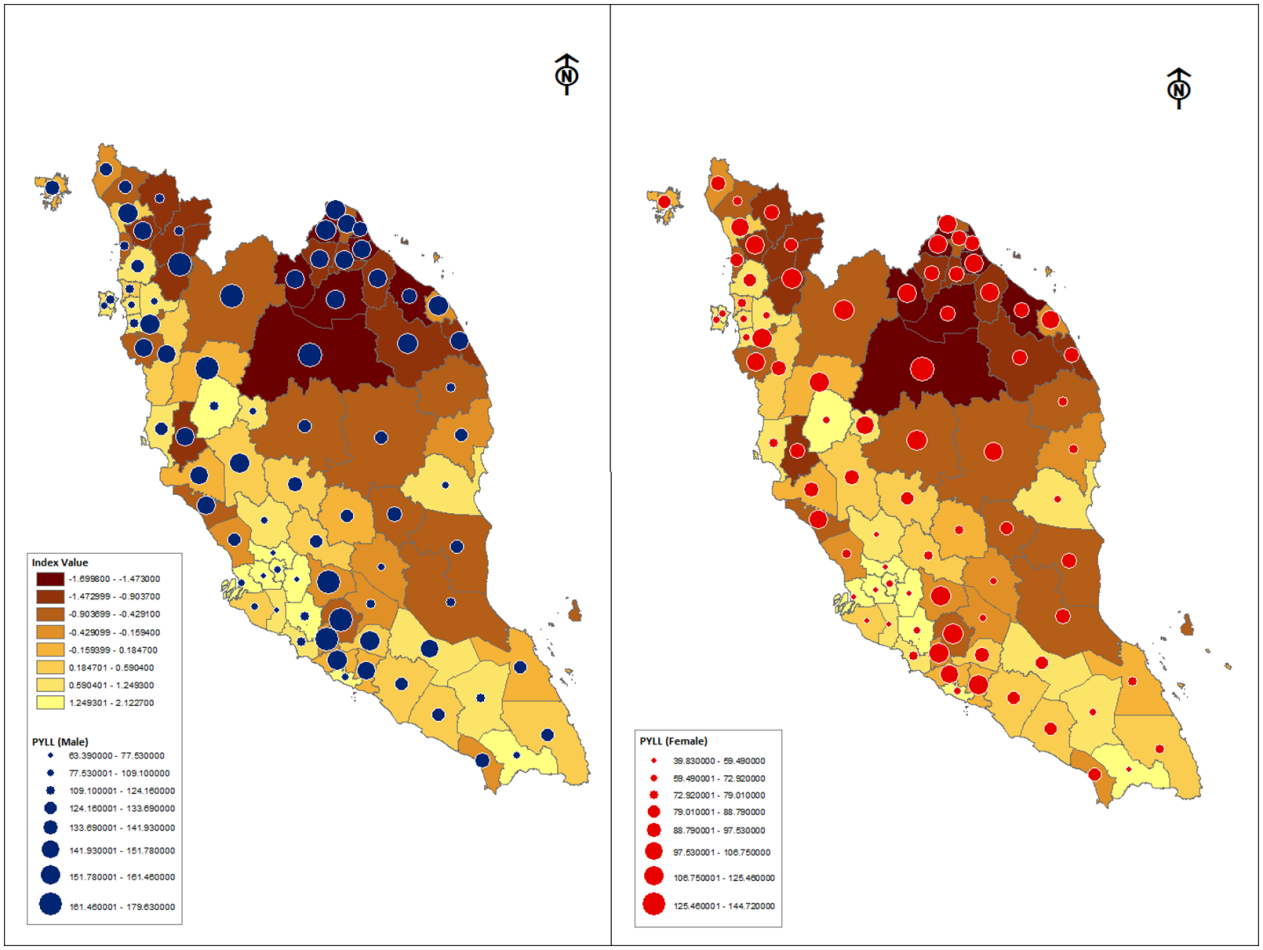
Geographical distribution of the Socioeconomic Index and Potential Years of Life Lost (PYLL) for males and females in Peninsular Malaysia. Yellow areas represent high socioeconomic advantage and dark brown areas, low socioeconomic advantage. The size of the blue and red circles represents the extent of premature mortality among males and females respectively. As values for PYLL differ for both genders, different ranges are used for both; therefore comparisons are for between males or females geographically and not for between males and females.

Fig 1 shows maps of Peninsular Malaysia’s districts for PYLL for males and females. The poorest districts are to be found in the north-eastern and east coast areas of the peninsular. In general these areas also bear the highest burden of premature mortality for both males and females.

Table 4 displays the concentration indices and the 95% confidence intervals for PYLL by ethnic groups. The negative values of the concentration indices and their respective confidence intervals confirm that premature mortality is disproportionately concentrated among the poorer districts for every ethnic group except for non-citizens.

**Table 4:** Concentration Indices and 95% Confidence Intervals for PYLL by Ethnicity for All-Cause Mortality.

	Overall		Male		Female	
Ethnicity	Concentration index	95% CI	Concentration index	95% CI	Concentration index	95% CI
Malay	-0.1208	-0.1510 to -0.0906	-0.1170	-.01464 to -0.0876	-0.1244	-0.1540 to -0.0947
Indigenous Natives	-0.3382	-0.4662 to -0.2102	-0.3181	-0.4308 to -0.2056	-0.3683	-0.5278 to -0.2088
Chinese	-0.1039	-0.1405 to -0.0673	-0.1139	-0.1557 to -0.0721	-0.0732	-0.1088 to -0.0375
Indian	-0.0681	-0.1025 to -0.0336	-0.0547	-0.1013 to -0.0081	-0.0561	-0.0954 to -0.0169
Others	-0.0573	-0.1068 to -0.0078	-0.0539	-0.0978 to -0.0101	-0.0444	-0.1153 to 0.0266
Non Citizens	0.0388	0.0063 to 0.0713	0.0670	0.1016 to 0.1695	0.0027	-0.0413 to 0.0466

**Fig 2:**
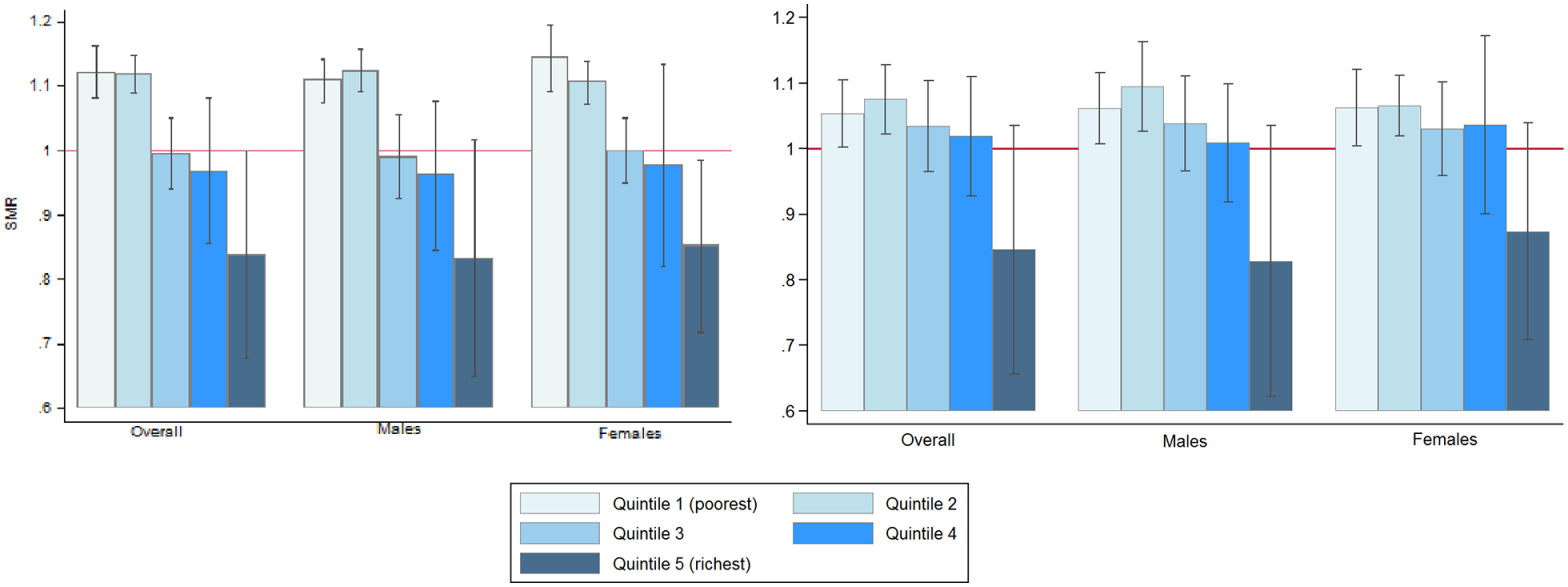
Age-gender adjusted SMRs and age-gender-ethnicity adjusted SMRs for each quintile by overall population, males and females in Peninsular Malaysia. The reference line at y = 1 indicates the SMR of the standard population, which is of Peninsular Malaysia.

Fig 2 shows SMRs by socioeconomic quintiles before and after adjustment for ethnic group distribution. The risk of dying is comparatively higher in the poorest two quintiles before ethnic group adjustment but with the exception of the richest quintile, the disparity disappears after adjusting for ethnic group. However, the resulting concentration index shows that inequality is still present in the age-gender-ethnicity adjusted SMR distribution (*C* = -0.0473, 95% CI (-0.0827 to -0.0119)).

## Discussion

This study presents a method to evaluate socioeconomic gradient in mortality using mortality data from national death registration system combined with data on socioeconomic status collected during population census. The findings show some evidence that a socioeconomic gradient in mortality had existed in Malaysia in 2000. This disparity was most marked in mortality among infants and children under the age of five years leading to a disparity in premature mortality. Although once adjusted for ethnicity, the gradient in mortality levels out, disparities still exists as majority of the population have worse mortality outcomes compared to the richest quintile.

Restrictions of our findings relate to the ecological nature of the study. Nevertheless, ecological studies such as this are useful to document and monitor inequalities in health by describing area-level deprivation and population health. Considering ecological fallacy, we are unable to directly extrapolate our findings to the level of individual health. The fact that districts have households which are spread across the socioeconomic distribution is acknowledged. However, the findings of this study are indicative that a gradient in mortality is likely to exist. The mortality differences from this study are yet to be inclusive of East Malaysia that consists of the states of Sabah and Sarawak which presumably may contribute to a greater level of inequality in mortality in Malaysia given their individual poverty rates of 8.1 and 2.4 respectively, which are far above the national average of 1.7 [20]. Furthermore, unconvincing administrative figures for mortality indicators such as the IMR and U5MR for the state of Sabah for the year 2012 which are as low as 3.3 and 4.3 [21] may be due to under-reporting, due to the exclusion of inaccessible populations and mobile rural communities [22]. With these spurious numbers included in the overall national mortality rate calculations, it is conjectured that mortality rates in Malaysia could be higher than reported.

With regard to child mortality, although national averages are low [23], better outcomes have been found among socioeconomically advantaged districts. These findings are coherent with the results of a study investigating disparities in under-5 mortality of 43 developing countries [24] which indicated increase in inequalities in under-5 mortality. In contrast, two studies done in Brazil and Indonesia [25,26] did not find a significant increase in socioeconomic inequalities in under-5 mortality. This was assumed to have resulted from the increasing level of education of mothers. Another study in Malaysia has looked at the association between material deprivation and relative risk of infant mortality [27]. The deprivation index that was utilised consisted of four variables; namely, percentage of households that do not own a car or house, no access to piped water and those in the labour force who are unemployed. The number of infant deaths was derived from only the year of interest. The conclusions were that areas that were deprived tended to have high risk of infant mortality. Although different methodology was used in our study, basically similar implications for infant mortality can be drawn.

Development and industrialisation in Peninsular Malaysia have been more prominent in the west coast compared to the east coast which previously depended heavily on agricultural means as a major source of income [28]. For that reason, the states (and their districts) of the west coast are more urbanised and wealthier compared to the east coast. Higher mortality rates in poorer districts are reflective of an environment with less opportunities or facilities for a better quality of life for households there. All districts regardless of average wealth of its population have groups of people who are disadvantaged and therefore susceptible to poorer health outcomes. However, if the district itself is wealthy, it will have better infrastructure, healthcare and social services which are able to alleviate the burden of disease across its population [29].

Generally, we found higher mortality reflected in districts with socioeconomically less advantaged households. These were districts which generally constitute of households that scored lowly on the socioeconomic index. The index, composed of household’s acquisition of assets, housing quality, access to utilities and infrastructure, and education level and employment status of household head are collectively representative of household’s socioeconomic position (SEP). Socioeconomic position strongly influences health behaviour, environmental exposure and health care [30]. SEP is also vital with regard to health inequities because of the extent that it can be modified by social, welfare and redistributive policies [31] which therefore makes it a favourable health policy as well [32].

Apart from SEP, a strong health system based on universal health coverage essentially contributes to fair consequences in the distribution of health. Currently, Malaysia operates a two-tier health care system which comprises of the government subsidised public health care and a coexisting user-charged private health care system which is paid for using household out-of-pocket payments, funding from employers (that provide employee medical benefits) and to a slighter extent, personal health insurance [33,34]. The public health system which provides universal health coverage for the entire population [35,36] has proven to have benefitted the whole population including the low and middle income groups who pay very low user fee to utilise health services. However with regard to private health care, besides the high charge for services there also appears to be a regional imbalance. The proliferation of private health care facilities which occurred during the 1990s is concentrated mostly in urban regions where the demand exist [37] [34]. There was a significant increase in private hospitals from 50 private hospitals with 1,171 beds in 1980 to 224 hospitals with 9,949 beds in 2001. Unlike public health care, private health care is mostly utilised by the rich [34].

Other findings of this study include differences in mortality rates between males and females. The higher rates among men compared to women in Malaysia is attributed to biological and behavioural factors [38]. Risky health behaviours such as smoking contribute largely to the deaths among men [39,40]. This may explain the higher deaths among Malaysian men as about 43.9% smoked a tobacco product compared to only 1.0% of women [41].

Malays were found to be highly concentrated in the poorer socioeconomic quintiles implying that they are relatively poorer than all other ethnic groups. This study also found that among Malaysian citizens, premature mortality is concentrated among the poor for not only the Malays but every ethnic group. This suggests that national policies should emphasise the degree of need rather than ethnic based policies to ensure that support is provided and distributed in an equitable manner. This is vital to prevent the gradient in health from becoming any steeper.

In conclusion, it is important for policy-makers to reflect and act on the indication that a pro-poor distribution in mortality is expected to exist in Malaysia despite decades of public policies which were established on the basis of equity. This upholds the necessity of stringent policy monitoring and evaluation and reconsideration of proposed policies that could further widen this disparity. Economic inequalities should foremost be addressed as they are known to be the key drivers of other dimensions of inequalities such as inequalities in health [42]. The concept of proportionate universalism should be incorporated in future policies as we work towards the SDG to reduce inequalities within the country.

## Supporting Information

S1 Appendix Distribution and justification of socioeconomic index. (DOCX)
