# Supplementary material for: Are the Poor Dying Younger in Malaysia? An Examination of the Socioeconomic Gradient in Mortality
Source: PLoS One. 2016 Jun 30;11(6):e0158685. doi: 10.1371/journal.pone.0158685 (PMC4928844; doi:10.1371/journal.pone.0158685)
Supplement: S1 Appendix — (DOCX) [file pone.0158685.s001.docx]

**S1 Table. Weights Assigned to Variables in the Socioeconomic Index**

| **Category** | **Description** | **Variable** | **Weight** |
| --- | --- | --- | --- |
| Quality of dwelling | Construction material of outer walls | Brick | 0.2772 |
|  |  | Brick & Plank & Others | -0.1359 |
|  |  | Plank | -0.209 |
|  | Number of bedrooms | Three or more rooms | 0.2099 |
|  |  | Two rooms | -0.1082 |
|  |  | Less than two rooms | -0.1764 |
| Access to utilities and infrastructure | Drinking water supply | Treated piped water | 0.1494 |
|  | Type of toilet facility | Flush system | 0.2865 |
|  | Garbage collection facility | Quarters | 0.2363 |
|  |  | Area | 0.0047 |
|  |  | None | -0.2556 |
| Durable consumer goods | Durable consumer goods | Video / VCD / DVD | 0.2425 |
|  |  | Mobile phone | 0.2277 |
|  |  | Fixed Telephone Line | 0.2215 |
|  |  | Personal Computer | 0.2079 |
|  |  | Washing Machine | 0.2073 |
|  |  | Air-Conditioner | 0.2054 |
|  |  | Refrigerator | 0.1942 |
|  |  | Microwave | 0.185 |
|  |  | Internet Subscription | 0.1746 |
|  |  | 2 or more Motorcars | 0.1547 |
|  |  | Television | 0.1512 |
|  |  | Radio or Hi-Fi set | 0.1427 |
|  |  | 1 Motorcar | 0.1387 |
|  |  | 2 or more Motorcycles | -0.0099 |
|  |  | 1 Motorcycle | -0.0477 |
|  |  | None | -0.1111 |
| House ownership | House ownership | Own this house or any other | 0.0046 |
| Education | Education level | Tertiary | 0.1377 |
|  |  | Upper/Post secondary | 0.0963 |
|  |  | Lower secondary | -0.0001 |
|  |  | Primary | -0.1011 |
|  |  | Never | -0.1271 |
| Employment | Employment status | Employed | 0.047 |

An in-depth literature review on wealth, asset or socioeconomic indices used in different countries around the world was carried out. Based on that, variables that are suitable and appropriate in the Malaysian context were short-listed. Frequencies of all variables were checked for clumping (≥ 97%) and truncation (≤ 3%). If that were the case, variables were not considered. All usable variables were then recoded to binary variables (0 or 1). The weight of each item is the factor score from the first principal component of the correlation matrix; using only information from the head of households. The percentage of variance explained by the first principal component is 18.97%. The literature points out that the proportion of variance explained by the first principal component is mostly between 12 to 27 percent [[1-3](#_ENREF_1)].

**S2 Table. Internal Coherence of Variables of the Socioeconomic Index**

| **Description** | **Variable** | **Q1 (Poorest)** | **Q5 (Richest)** |
| --- | --- | --- | --- |
| Construction material of outer walls | Brick | 29.1 | 88.0 |
|  | Plank | 44.0 | 4.7 |
| Number of bedrooms | Three or more rooms | 45.0 | 70.1 |
|  | Less than two rooms | 20.1 | 4.2 |
| Drinking water supply | Treated piped water | 78.9 | 99.2 |
| Type of toilet facility | Flush system | 27.7 | 90.2 |
| Garbage collection facility | Quarters | 11.1 | 64.8 |
|  | Area | 22.7 | 29.2 |
|  | None | 66.2 | 6.0 |
| Durable consumer goods | Video / VCD / DVD | 34.0 | 77.8 |
|  | Mobile phone | 10.9 | 52.9 |
|  | Fixed Telephone Line | 48.0 | 77.6 |
|  | Personal Computer | 6.6 | 28.9 |
|  | Washing Machine | 61.6 | 81.1 |
|  | Air-Conditioner | 5.5 | 29.7 |
|  | Refrigerator | 76.4 | 91.8 |
|  | Microwave | 6.6 | 27.6 |
|  | Internet Subscription | 2.4 | 16.5 |
|  | 2 or more Motorcars | 4.6 | 19.4 |
|  | Television | 88.4 | 96.0 |
|  | Radio or Hi-Fi set | 79.2 | 90.2 |
|  | 1 Motorcar | 34.2 | 53.6 |
| Education level | Tertiary | 5.9 | 21.3 |
|  | Never | 19.6 | 4.7 |

Values shown here are percentages.Percentages may not total to 100% due to rounding.

S3 Table describes the characteristics of household heads by socioeconomic quintiles.

**S3 Table. Characteristics of household heads (n=77,460) by socioeconomic quintiles**

|  |  | Quintiles (poor to rich), % | | | | |
| --- | --- | --- | --- | --- | --- | --- |
|  | Total, n | 1 | 2 | 3 | 4 | 5 |
|  |  | (n=15,652) | (n=15,837) | (n=16,334) | (n=14,429) | (n=15,208) |
| **Area** |  |  |  |  |  |  |
| Urban (≥10,000 population) | 51,617 | 28.3 | 45.0 | 71.6 | 94.0 | 97.4 |
| Rural (< 10,000 population) | 25,843 | 71.7 | 55.0 | 28.4 | 6.1 | 2.6 |
| **Sex** |  |  |  |  |  |  |
| Male | 66,664 | 84.3 | 84.8 | 87.4 | 85.9 | 87.9 |
| Female | 10,796 | 15.7 | 15.2 | 12.6 | 14.2 | 12.1 |
| **Age, Median (IQR)** | 44  (35 – 54) | 47  (38 - 58) | 47  (37 - 58) | 43  (35 -53) | 42  (33 - 52) | 41  (33 - 50) |
| **Ethnicity** |  |  |  |  |  |  |
| Malay Bumiputera | 44,290 | 87.3 | 63.3 | 51.7 | 38.9 | 43.1 |
| Other Bumiputera | 778 | 1.3 | 1.1 | 1.0 | 1.0 | 0.7 |
| Chinese | 21,934 | 6.7 | 24.7 | 30.3 | 42.9 | 38.4 |
| Indians | 6,779 | 1.6 | 6.9 | 12.5 | 10.4 | 12.5 |
| Others | 436 | 1.0 | 0.2 | 0.4 | 0.6 | 0.6 |
| Non-citizens | 3,243 | 2.2 | 3.7 | 4.2 | 6.2 | 4.8 |
| **Marital status** |  |  |  |  |  |  |
| Never married | 6,574 | 4.2 | 6.2 | 7.6 | 13.1 | 11.9 |
| Married | 63,217 | 82.0 | 81.4 | 83.9 | 78.9 | 81.6 |
| Widowed | 6,640 | 11.7 | 11.1 | 7.5 | 6.8 | 5.5 |
| Divorced/ Separated | 1,029 | 2.1 | 1.2 | 1.0 | 1.3 | 1.0 |
| **Sector** |  |  |  |  |  |  |
| Government | 9,285 | 19.7 | 16.3 | 15.9 | 14.9 | 14.1 |
| Private | 26,759 | 23.9 | 37.6 | 53.2 | 59.8 | 61.0 |
| Own business | 21,201 | 56.4 | 46.1 | 31.0 | 25.3 | 24.9 |

Socioeconomic quintiles are population-weighted, using census totals from each district. Household heads described here are from the data used for the socioeconomic index; 2% sample of the year 2000 Population and Housing Census of Malaysia. The variable, sector, excludes household heads who are outside the labour force, unemployed or with unknown information. Percentages may not total to 100% due to rounding.

Urban districts were mostly grouped in the richer end of the quintiles and rural districts were mostly grouped in the poorer end of the quintiles. 94% and 97.4% of household heads in quintile 4 and 5 respectively were from urban areas; whereas almost 72% of household heads in quintile 1 were from rural areas. The percentage of household heads that are female increased from the richest quintile (12.1%) to the poorest quintile (15.7%). The median age of the household heads also increased from 41 years for the richest quintile to 47 years in the poorest quintile; however, there is an overlap in the interquartile range for all quintiles. Concerning ethnicity, Malay Bumiputeras, other Bumiputeras (indigenous people), and Others have the highest proportion in the poorest quintile compared to all other quintiles. This is unlike the Chinese, Indians and non-citizens who all have the lowest proportion in the poorest quintile as compared to all other quintiles. Households with heads who were widowed, divorced or separated also had the highest proportion in the poorest quintile. Households with heads who work in the private sector have the highest percentages in quintiles 3 to 5 compared to heads who have their own businesses who have the highest percentages in quintiles 1 and 2.

S1 Fig shows the histograms of the standardised socioeconomic index by states in Peninsular Malaysia.

The distribution of the socioeconomic index is right-skewed for the Northern-eastern states (Kelantan, Terengganu and Perlis) which mean a lower average value of the index compared to the other states. The states in the west coast (Kuala Lumpur, Selangor, Pulau Pinang, Johor, Negeri Sembilan and Perak) have left-skewed distributions, meaning a high average value of the index. The distribution of the index for the other states (Melaka, Kedah and Pahang) is generally balanced

**
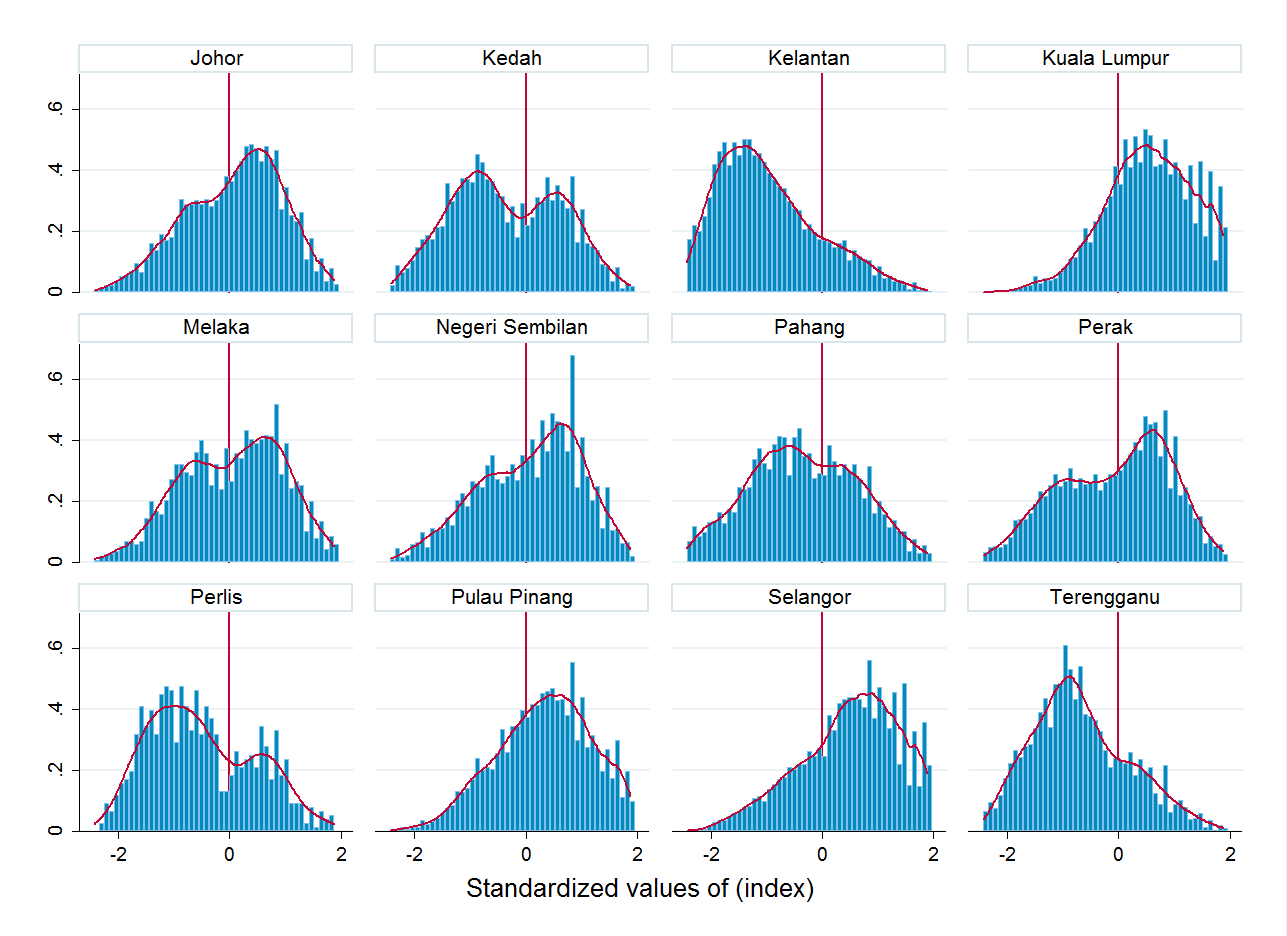
**

**S1 Fig. Distribution of socioeconomic index by states in Peninsular Malaysia**

For justification of the adaptation of the socioeconomic index as a proxy for household wealth to characterise SEP, the similarity of the distribution of the mean of the index (S2 Fig) is compared to the national figures of mean income for states (S3 Fig). The similarity of both distributions justifies the use of the socioeconomic index as a proxy measure for income. The map of the states of Peninsular Malaysia is shown in S4 Fig. A study has been conducted in Malaysia [[4](#_ENREF_4)] looking at deprived areas of Peninsular Malaysia. However, the study utilised a deprivation index which looked at percentage of households in a district without a particular asset/household item or risk of stillbirths, neonatal and infant mortality. This is different from ours where a socioeconomic index was used as a measure of household wealth.


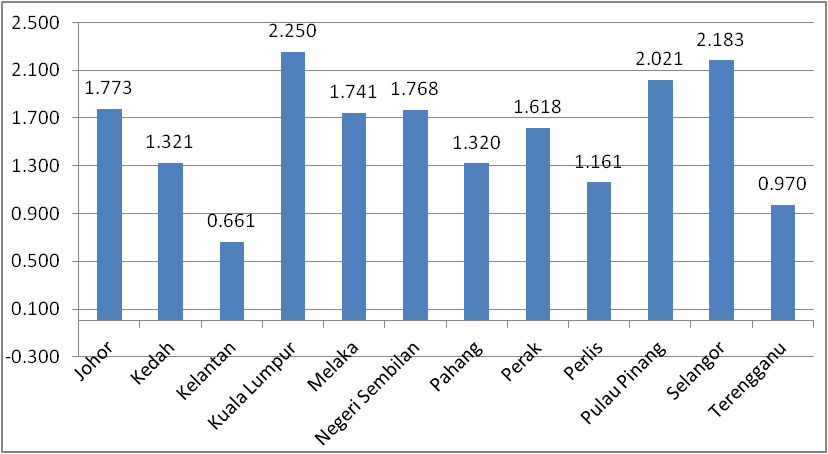


S2 Fig. Mean of socioeconomic index state, Peninsular Malaysia, 2000

S3 Fig. Mean monthly household income (MYR) by state, Peninsular Malaysia, 1999 & 2002 [[5](#_ENREF_5)]


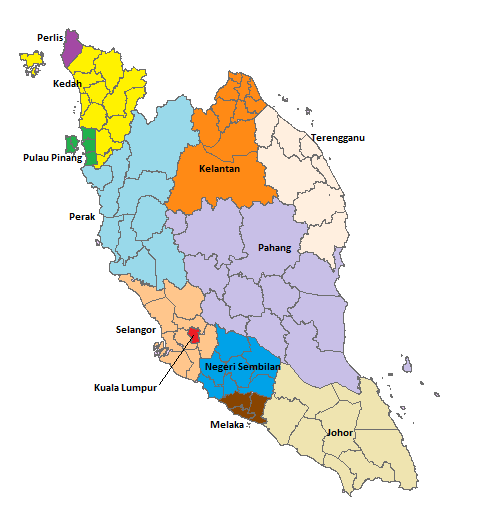


S4 Fig. Map of the States of Peninsular Malaysia

**References**

1. Vyas S, Kumaranayake L (2006) Constructing socio-economic status indices: how to use principal components analysis. Health policy and planning 21: 459-468.

2. Filmer D, Pritchett LH (2001) Estimating wealth effects without expenditure Data—Or tears: An application to educational enrollments in states of india*. Demography 38: 115-132.

3. Houweling TA, Kunst AE, Mackenbach JP (2003) Measuring health inequality among children in developing countries: does the choice of the indicator of economic status matter? International journal for equity in health 2: 8.

4. Fam S-F, Ismail N, Jemain AA, Melaka UTM (2014) Where are Peninsular Malaysia's most deprived areas? Regional Statistics Conference 2014 Kuala Lumpur.

5. Economic Planning Unit (2012) Mean Monthly Gross Household Income by Ethnicity, Strata and State, Malaysia, 1970-2012. In: Statistics HIP, editor.
